# Mental wellbeing and quality of life in prostate cancer (MIND-P): Protocol for a multi-institutional prospective cohort study

**DOI:** 10.1371/journal.pone.0284727

**Published:** 2023-04-24

**Authors:** Oliver Brunckhorst, Jaroslaw Liszka, Callum James, Jack B. Fanshawe, Mohamed Hammadeh, Robert Thomas, Shahid Khan, Matin Sheriff, Hashim U. Ahmed, Mieke Van Hemelrijck, Gordon Muir, Robert Stewart, Prokar Dasgupta, Kamran Ahmed

**Affiliations:** 1 MRC Centre for Transplantation, Guy’s Hospital Campus, King’s College London, King’s Health Partners, London, United Kingdom; 2 Department of Urology, Queen Elizabeth Hospital, Lewisham and Greenwich NHS Trust, London, United Kingdom; 3 The Primrose Oncology Unit, Bedfordshire Hospitals NHS Foundation Trust, Bedford, United Kingdom; 4 Department of Urology, East Surrey Hospital, Surrey and Sussex Healthcare NHS Trust, Redhill, United Kingdom; 5 Department of Urology, Medway NHS Foundation Trust, Gillingham, United Kingdom; 6 Imperial Prostate, Division of Surgery, Department of Surgery and Cancer, Imperial College London, London, United Kingdom; 7 Imperial Urology, Charring Cross Hospital, Imperial College Healthcare NHS Trust, London, United Kingdom; 8 Translational Oncology and Urology Research (TOUR), School of Cancer and Pharmaceutical Sciences, King’s College London, London, United Kingdom; 9 Department of Urology, King’s College Hospital NHS Foundation Trust, London, United Kingdom; 10 King’s College London Institute of Psychiatry, Psychology and Neuroscience, London, United Kingdom; 11 South London and Maudsley NHS Foundation Trust, London, United Kingdom; 12 Department of Urology, Guy’s Hospital, Guy’s and St Thomas’ NHS Foundation Trust, London, United Kingdom; 13 Department of Urology, Sheikh Khalifa Medical City, Abu Dhabi, United Arab Emirates; 14 Khalifa University, Abu Dhabi, United Arab Emirates; The Hong Kong Polytechnic University, HONG KONG

## Abstract

**Background:**

The mental wellbeing implications of a prostate cancer diagnosis are increasingly being realised. Significant mental health symptoms such as depression and anxiety, along with related constructs such as fear of cancer recurrence, body image and masculine self-esteem issues are prevalent. However, less is understood about potential prognostic factors for these outcomes in prostate cancer patients. Therefore, this study aims to primarily explore potential treatment, patient and oncological factors associated with mental wellbeing outcomes in the initial prostate cancer follow-up period.

**Methods:**

MIND-P is a multi-institutional prospective cohort study recruiting newly diagnosed prostate cancer patients for 12-month follow up. It will aim to recruit a final sample of 300 participants undergoing one of four treatment options: active surveillance, radical prostatectomy, radical radiotherapy, or hormone monotherapy. Questionnaire-based data collection consists of multiple validated mental, physical, and social wellbeing outcomes at baseline and 3-monthly intervals until study completion. Primary analysis will include evaluation of treatment undergone against multiple mental wellbeing outcomes. Secondary analysis will additionally explore multiple patient and oncological prognostic factors of potential importance, along with the cumulative incidence of these outcomes, symptom trajectory and their association with subsequent functional and social outcomes.

**Conclusion:**

This cohort study aims to add to the existing limited literature evaluating significant prognostic factors for multiple mental wellbeing outcomes in newly diagnosed prostate cancer patients. This may be of potential use for guiding future prognosis research and of clinical use for identifying individuals potentially requiring additional surveillance or support during routine cancer follow up.

**Study registration:**

This study was prospectively registered on ClinicalTrials.gov (NCT04647474).

## Introduction

Prostate cancer represents a large global public health problem, accounting for almost 1.4 million new cases yearly and being the second most common cancer diagnosis in men [1]. This large incidence, combined with its increasing survival rates, means that not only are growing numbers being diagnosed, but more men are now living with and beyond their disease [2]. Due to this, there is a growing realisation that living longer, and surviving disease, does not always equate to living well. With this in mind survivorship care in cancer has become an increasingly acknowledged topic with large initiatives globally, including the National Cancer Survivorship Initiative (NCSI) in the United Kingdom and similar programmes across the United States such as from the American Cancer Society (ACS) and the American Society of Clinical Oncology (ASCO) [3, 4].

When considering the impact of disease and treatment on prostate cancer patients long term wellbeing, the physical consequences, including urinary and sexual dysfunction, are well described [5]. However, it is only more recently that the mental wellbeing impact of disease is becoming recognised [6]. Mental health issues, particularly depressive and anxiety symptoms are prevalent in this group affecting an estimated 17% of individuals and translating into high suicidal ideation and mortality [7]. Additionally, other issues outside of pure mental health symptoms appear to be important in these men. This includes fear of cancer recurrence (FCR) or progression which has previously been labelled as one of the most common unmet cancer needs [8]. Along with Prostate-Specific Antigen (PSA) Anxiety, FCR is a prevalent issue in patients with prostate cancer [9]. Furthermore, issues surrounding masculinity, masculine-self-esteem and body image appear important in this population and represent constructs which assess the concept of self-worth representing the view of our body and mind and how it is perceived by others around us [10]. All these issues are of undeniable significance in prostate cancer care, not only in their prevalence, but also on the impact they have on treatment choices, functional outcomes and even mortality [11, 12].

However, within much of the prostate cancer literature, physical outcomes after diagnosis and treatment remains the focus, despite not being the end-all of quality of life for patients [6]. This means that whilst it is clear mental wellbeing is an important factor, less appears to be understood about prognostic factors which are associated with poorer mental wellbeing. Beyond hormone therapy, which has been acknowledged as an important factor for outcomes such as depression, there is little consensus on other patient, oncological or treatment factors which are important in mental wellbeing outcomes [13–15]. This is particularly true for outcomes that fall outside of pure mental health, such as anxiety and depression. Additionally, where evidence does exist, including prognostic factors such as younger age, treatments undergone and oncological stage, this evidence is limited to general cancer populations [16–18]. However, it is not clear whether these translate within the prostate cancer setting, particularly when considering the unique and broad experiences these men have post diagnosis.

A better understanding of these factors is important, allowing for better counselling of patients, identification of those who require closer surveillance and better support to improve patient outcomes and gives targeted investigating causes for future research [19]. Additionally, having a good understanding of prognostic factors is vital when considering candidate predictors to use when developing subsequent multivariable prognostic models which may be subsequently used to risk stratify patients at the outset of follow up [20].

## Study aims

With the current literature deficiencies and limited existing evidence on prognostic factors for mental wellbeing outcomes in prostate cancer, the proposed study is primarily aimed as an exploratory study to further define potential factors of interest [19]. Therefore, the primary aim of the study is to evaluate the association between prostate cancer patients undergoing different management options and subsequent mental wellbeing outcomes in the initial cancer follow-up stage. We hypothesise there to be significant differences between different management groups in view of their varying physical symptom loads and demands. More specifically, that those undergoing hormone monotherapy possess the poorest mental wellbeing outcomes, with those undergoing active surveillance the least.

In addition to this, secondary aims include:

1. To explore patient and oncological prognostic factors associated with mental wellbeing outcomes in patients with prostate cancer.
2. Define incidence of individual mental wellbeing issues in the first year after diagnosis.
3. Explore trajectory of mental wellbeing symptoms in the first year after diagnosis.
4. Assess the relationship between mental wellbeing symptoms and functional, social wellbeing and general health outcomes.

We hypothesise that several patient factors including age, previous mental health history and comorbidities will be significant prognostic factors for poorer mental wellbeing outcomes, based on existing literature in other cancer groups. Similarly, we hypothesise that oncological factors such as stage at presentation and time since diagnosis will be significantly associated with mental wellbeing. Specifically, for time since diagnosis/ symptom trajectory, we hypothesise a downward trend of symptom severity for symptoms such as depression, anxiety and FCR due to increasing time since original diagnosis and reduced uncertainty present post treatment, whilst an upwards trajectory for body image and masculinity symptoms is expected as treatment related side-effects begin to increase with time. Lastly, in view of the extensive literature linking mind and body outcomes in various urological conditions, we believe those with significant mental wellbeing symptoms will possess poorer functional outcomes at study conclusion.

## Materials and methods

### Study design and setting

The Mental wellbeIng aND quality of life in Prostate cancer (MIND-P) study is a prospective and longitudinal cohort study following newly diagnosed prostate cancer patients for 12 months from diagnosis. MIND-P is a multi-institutional study across eight secondary and tertiary hospital sites across London and the south of the United Kingdom (UK). Participating sites are listed in Table 1. Study recruitment started in January 2021, and we expect that the last participant will finish the study in Spring/Summer 2023.

**Table 1:** Participating study sites and locations.

Hospital Site	Hospital Trust	Location
King’s College Hospital	King’s College Hospital NHS Foundation Trust	Camberwell, London, UK
Princess Royal University Hospital	King’s College Hospital NHS Foundation Trust	Orpington, UK
Guy’s Hospital	Guy’s and St Thomas’ NHS Foundation Trust	Southwark, London, UK
Charring Cross Hospital	Imperial College Healthcare NHS Foundation Trust	Hammersmith, London, UK
Queen Elizabeth Hospital	Lewisham and Greenwich NHS Trust	Woolwich, London, UK
East Surrey Hospital	Surrey and Sussex Healthcare NHS Foundation Trust	Redhill, UK
Medway Maritime Hospital	Medway NHS Foundation Trust	Gillingham, UK
Bedford Hospital	Bedfordshire Hospitals NHS Foundation Trust	Bedford, UK

### Population

The study population consists of newly diagnosed prostate cancer patients undergoing one of four management options for their disease, including:

- Active Surveillance
- Radical Prostatectomy
- Radical Radiotherapy
- Hormone Monotherapy

Full inclusion criteria for the study are:

- Adults (>18 years old) males with no limit on upper age
- Possess a new diagnosis of histologically proven or clinically likely prostate cancer with no limits on grade, stage, or histological type of prostate cancer
- Post multi-disciplinary team discussion with allocation of a suggested management strategy
- Due to undergo one of the previously listed management options
- Follow up undertaken by urology, oncology, or mixed uro-oncology teams

Exclusion Criteria for the study are:

- Patient has already undergone the allocated intervention
  ○ Attended second active surveillance follow up
  ○ Post-surgery
  ○ Post first radiotherapy dose
  ○ Received >1 dose (initial dose) of Gonadotropin-releasing hormone agonist/antagonist or more than 3 months of daily tablets.
- Patients receiving the following managements:
  ○ Palliative patients on symptom control only
  ○ Patients allocated to watchful waiting
  ○ Initially due to undergo any type of Focal therapy e.g., high intensity focused ultrasound (HIFU)
  ○ Patients receiving adjuvant combination therapy e.g., chemotherapy pre radiotherapy or surgery (with the exclusion of hormone therapy)
  ○ Metastatic patients undergoing chemotherapy alone
- Patients presenting with recurrence or progression of prostate cancer
- Concurrent management for another cancer diagnosis
- Recent admission to an inpatient psychiatric facility within the previous 12 months prior to diagnosis of prostate cancer
- Patients lacking capacity to consent or undertake in the research
- Those unable to complete the required surveys, such as those not able to understand English or those with severe learning disability

### Study procedures

#### Recruitment

**Fig 1:**
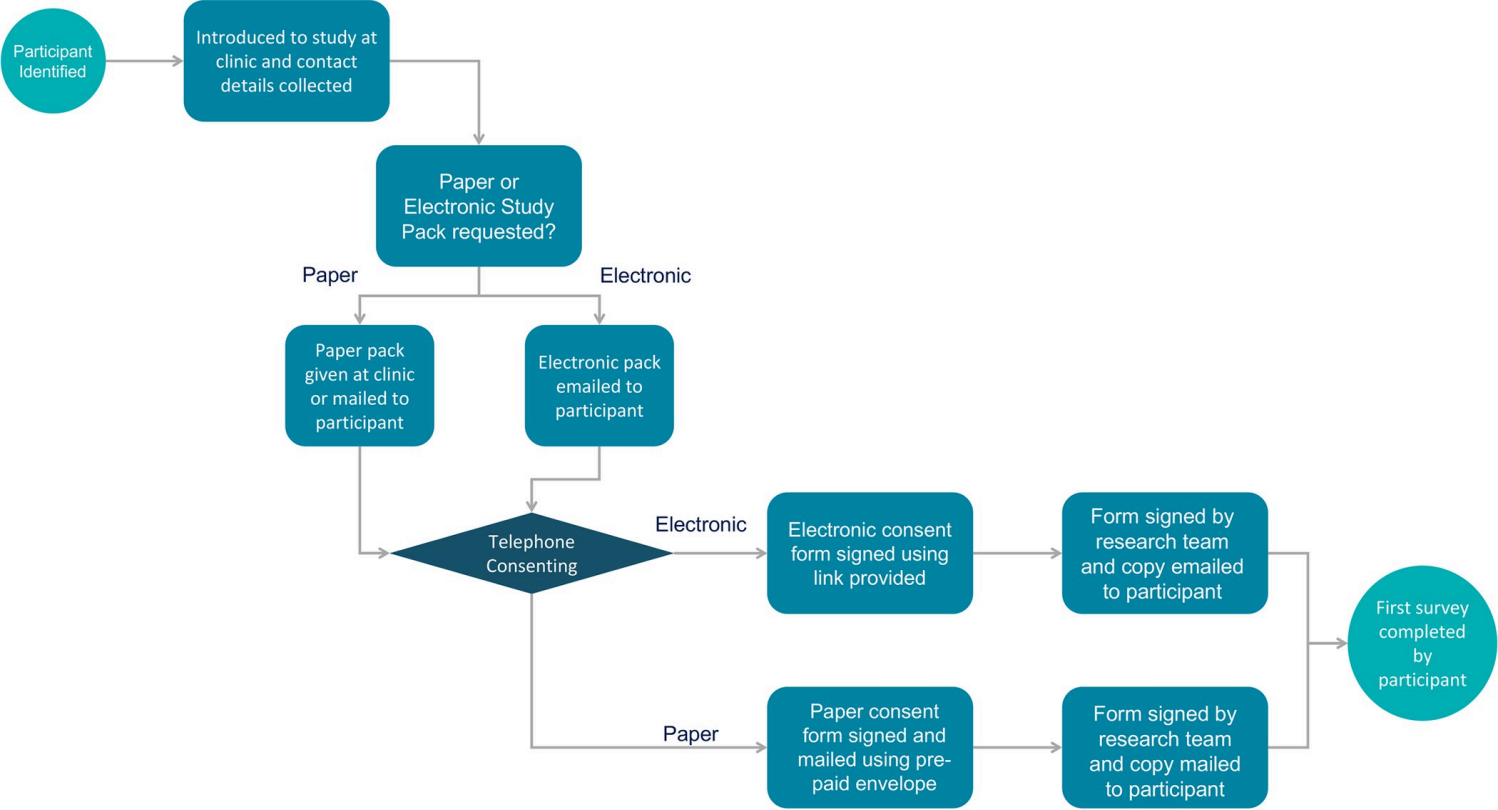
Participant recruitment and consenting procedure overview.

Identification of potentially eligible individuals is conducted at one of the participating sites of the study. Suitable patients are screened against inclusion and exclusion criteria through local outpatient clinic lists or multi-disciplinary team meeting lists. Potential participants are subsequently asked if they are interested in participating and if they are happy to be contacted by the research team centrally. If interested, contact details are subsequently passed on and participants receive either paper or electronic study packs, depending on their preference. These contain an extensive participant information sheet along with either a physical, or a link for a consent form (S1 File). Subsequently post receipt of the study pack a member of the research team contacts the individual via telephone to conduct a full informed consenting process using a consenting proforma. If individuals are subsequently happy to participate, they are asked to either complete the physical consent form and return this to the research team using a pre-paid envelope or complete the online consent form. A signed copy is subsequently returned to the individual. Individuals then complete their baseline questionnaires. The full process is summarised in Fig 1. This hybrid and remote consenting process was chosen to ensure minimal contact with participants during the COVID-19 pandemic, whilst allowing for access to those without the ability or desire to conduct this electronically

## Data collection and outcomes

**Fig 2:**
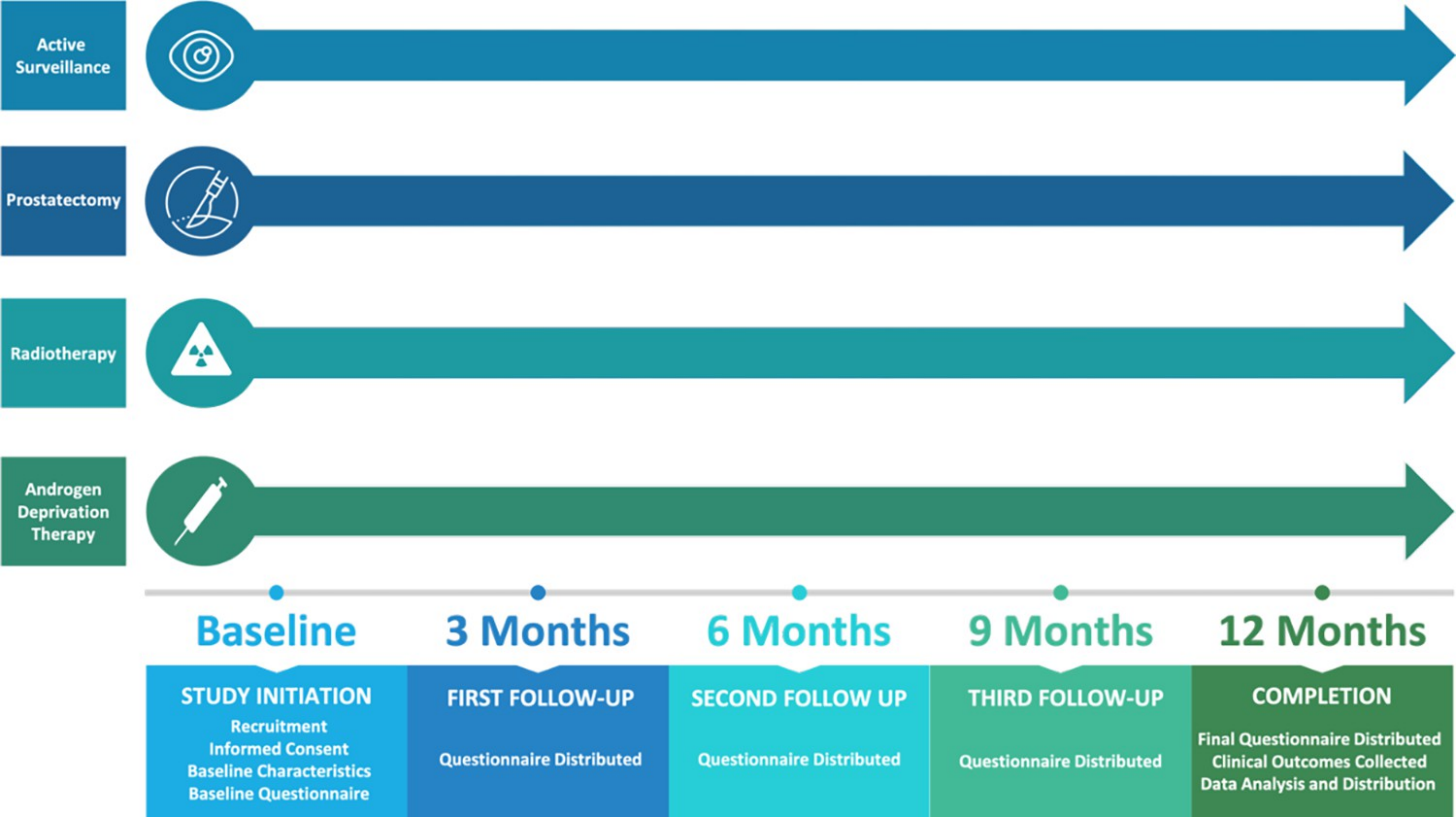
Study timeline.

This questionnaire-based study is conducted remotely, with no physical follow ups within a hospital site. Again, this design was selected to minimise patient risk during the COVID-19 pandemic. Recruited individuals are asked to complete a series of online or postal questionnaires depending on participant preference (S2 File). Participants are requested to complete the same questionnaire containing a battery of validated mental, physical, and social wellbeing measures at baseline, 3-, 6-, 9-, and 12-months (Fig 2). In addition, at baseline participants are requested to complete some demographic information. Reminders are sent to participants at two- and four-weeks post questionnaire dates if not received within this time frame. Participants are then assumed to be no longer interested in participation and marked as lost to follow up if not returned thereafter. Clinical data extraction is also conducted by the research team to collect baseline disease characteristics and subsequent clinical outcomes. A full list of variables to be collected and analysed as exposure/independent variables are included in Table 2. End of study participation will be at 12 months upon completion of the final questionnaire.

**Table 2:** Exposure variables collected for subsequent prognostic evaluation during the study.

**Patient Factors**	Age at diagnosis, ethnicity, relationship/co-occupancy status, employment status, smoking status, alcohol intake, index of multiple deprivation, medical co-morbidities, current or previous mental health history, family history of mental health conditions and family history of prostate cancer
**Oncological Factors**	Gleason grade and ISUP classification, number of positive biopsy cores, risk stratification, PSA at diagnosis, TNM Score and stage classification.
**Treatment Factors**	Final treatment received, presence of positive surgical margins

Primary study outcomes collected within questionnaires included validated psychometric measures for mental wellbeing post prostate cancer diagnosis. Important mental wellbeing outcomes were defined through multiple extensive and systematic searches of the existing literature due to variable current definitions [7, 9, 10]. This selected five mental wellbeing constructs of interest which will be included within the overall primary outcome of ‘mental wellbeing’:

- Depressive Symptoms
- Anxiety Symptoms
- Fear of Cancer Recurrence/Progression
- Body Image Perception
- Masculine Self-Esteem

Selection of subsequent validated psychometric measures for these outcomes was based on the most utilised tools, or those with the best psychometric properties within a prostate cancer population. These were defined by previous reviews or through a dedicated review for body image and masculinity outcomes [7, 9, 21]. Final psychometric scales selected were:

- Patient Health Questionnaire (PHQ)-9—Depression symptoms [22]
- Generalised Anxiety Disorder (GAD)-7 –Anxiety symptoms [23]
- Fear of Recurrence Scale 7 (FCR7)—Fear of cancer recurrence/progression [24]
- Body Image Scale (BIS)–Body image perception [25]
- Prostate Cancer-Quality of Life (PC-QOL) Masculine Self-Esteem Subset–Masculinity [26]

Importantly, the FCR7 scale items wording was adapted to ensure both recurrence and progression fear was evaluated to ensure ability of both baseline evaluation, and that of individuals not due to undergo curative treatments (e.g., active surveillance or hormone monotherapy). Similarly, the final item of the BIS (dissatisfaction with scar) was removed to ensure consistent evaluation between treatment groups. Internal consistency of these will be evaluated at conclusion through Cronbach’s alpha values to ensure they represent valid measures of these outcomes within the population.

Additional outcomes collected within questionnaires were validated measures of social, physical, and general health including:

- The Expanded Prostate Cancer Index Composite-26 (EPIC-26)–Functional/Physical Symptoms
- Functional Assessment of Cancer Therapy–General (FACT-G) Social Wellbeing Subscale–Social Wellbeing
- Short Form– 12 (SF12)–Impact of disease on health

These outcomes will be utilised as both dependent and exposure variables depending on the subsequent analysis conducted.

### Statistical analysis

Descriptive statistics will be calculated for the study population and individual study outcomes. Important definitions to be utilised throughout the data analysis are the threshold set for significant mental wellbeing symptoms in each of the measures collected. For depressive or anxiety symptoms a score of 10 or greater on the PHQ-9 or GAD 7 scales will be used for depression and anxiety respectively, representing moderate symptoms and has been demonstrated to correlate well to depressive or anxiety disorders diagnoses [23, 27]. For the FCR7 scale, a value of 17 or greater was selected, being representative of moderate fear of recurrence/progression as per the tool’s original development [24]. For the BIS, a cut of 10 or greater was selected as a previously demonstrated useful clinically relevant cut off point [28]. Finally, no clear-cut offs exist for the PC-QOL masculine self-esteem subset specifically. However, previously the tool authors have utilised scores of less than 60 to signify a low score for other subsets of the scale, and hence this was selected for this study [29].

Missing data during study follow-up through participant loss to follow up is expected, impacting subsequent analyses for primary and secondary outcomes. Where possible, multiple imputation will be considered for missing outcome data, with complete case analysis to be used for analyses where this is not possible.

### Primary outcome

Along with the primary objective, the primary study outcome will be the unadjusted mean differences between different management groups of each individual mental wellbeing outcome at 3-, 6-, 9- and 12-months post diagnosis. For this a one-way ANOVA between treatment groups, is planned at each time frame. Subsequent Tukey’s Honest Significant Difference post-hoc analysis will be conducted on any means identified to be statistically significantly different.

### Secondary outcomes

The following outcomes are not accounted for within the sample size calculations and are therefore considered exploratory. Each planned analysis will be divided per secondary aim of the study.

1. For exploration of prognostic factors of mental wellbeing outcomes, baseline patient, oncological and treatment factors previously described will be evaluated against the binary outcome of the development of significant individual mental wellbeing symptoms during study follow up. For this analysis an initial univariable, followed by a multivariable regression analysis of potentially significant individual prognostic factors is planned.
2. The cumulative incidence of developing significant individual mental wellbeing outcomes during the 12-month study follow up will be calculated and presented as a percentage.
3. To evaluate the trajectory of mental wellbeing symptoms over a 12-month period and effect of time on symptom scores, mental wellbeing outcome scores of whole cohort and individual treatment cohorts will be evaluated at each follow up time. Evaluation of these scores will be conducted using multilevel mixed-effects analyses to account for the effect of recruitment site clustering and expected loss to follow up during the study.
4. The relationship between each individual mental wellbeing symptom and functional, social, and general health outcomes will be evaluated. Functional outcomes will include sexual, urinary and bowel symptoms from EPIC-26. Social wellbeing will include FACT-G subscale scores with general health defined using the SF-12 physical component score. All outcomes will be evaluated at 12-months. For this each mental wellbeing symptom will be continuously evaluated against these outcomes using univariable and subsequent multivariable linear regression, adjusted for known factors for functional and social outcomes.

### Sample size

Target sample size calculations are based on the primary aim and outcome of the study evaluating mean differences between different treatment cohorts. This was conducted for hypothesis testing against minimal clinically significant difference (MCSD) of the five mental wellbeing measures of interest, as previously reported in the literature. This was calculated utilising a one-way ANOVA across the four cohorts. Common standard deviation (SD) derived from the literature, or previous pilot data from a small cross-sectional sample were also used to subsequently calculate the effect size f value, with a power of 0.8 and an alpha value of 0.05 utilised. G*Power Software V3.1 [30] was used to calculate the required sample sizes. The largest required sample size across the five mental wellbeing measures was used as the required minimal sample size.

- PHQ-9 –MCSD of 5 [31], SD of 3.49 (pilot data)
- GAD 7 –MCSD of 5 [32], SD of 3.49 (pilot data)
- FCR7 –MCSD of 10, SD 7.01 [24]
- BIS–No clear MCSD published in literature [33], use of 10 as cut off for significant symptoms [25], SD of 3.93 (pilot data)
- Masculine Self-Esteem MCSD of 9.8 [34], SD of 15.79 [35]

Sample size calculations utilising the above values revealed a maximum requirement of 232 participants (58 per cohort) using the PC-QOL Masculine Self-Esteem Scale. Assuming a reasonable dropout rate of 25%, a requirement of 75 participants per cohort was set to give an overall target sample of 300 participants. During recruitment, individual treatment cohorts of these sizes will be targeted, with a gradual stop in recruitment across these different cohorts to gain sizes of groups as close to equal as possible. However, due to likely unequal dropout rates and some crossover to different treatment arms during follow up (e.g. those undergoing surveillance subsequently undergoing radical treatment) it is likely some disparity in cohort sizes will exist. These would subsequently require consideration within analysis and conclusions drawn from study results if any individual cohort contains fewer than the minimum sample size described above.

### Data storage and retention

All data is handled in accordance with current legislation, including General Data Protection Regulation (GDPR) and the Data Protection Act 2018. All data collected either digitally or physically is subsequently digitally stored in secure encrypted storage devices with password protected access. Physical data collected such as postal questionnaires is stored in locked file cabinets in areas with limited access. All digital data is pseudonymised with any identifiable data required for study conduct stored separately with only essential central research team having access to these. The study chief investigator (KA) acts as the overall responsible data controller for the study duration.

Following study completion all identifiable information will be destroyed following one year, once dissemination of results to participants has been completed. Subsequent storage of fully anonymised data and metadata arising from the study will be archived on the King’s College research depository for a minimum of 20 years and made publicly available through a unique DOI post publication of results. Express written consent for this long-term storage is sought from participants during recruitment.

### Ethics approval and safety considerations

The study abides by the principles of the Declaration of Helsinki with the study protocol and associated study documents receiving full prospective National Health Service (NHS) ethics committee and Human Research Authority (HRA) approval on 9^th^ November 2020 (NHS REC Reference: 20/LO/1136). Additionally, local institutional approval at each individual study site was gained prior to initiation of recruitment. MIND-P is also registered on ClinicalTrials.gov (NCT04647474).

**Fig 3:**
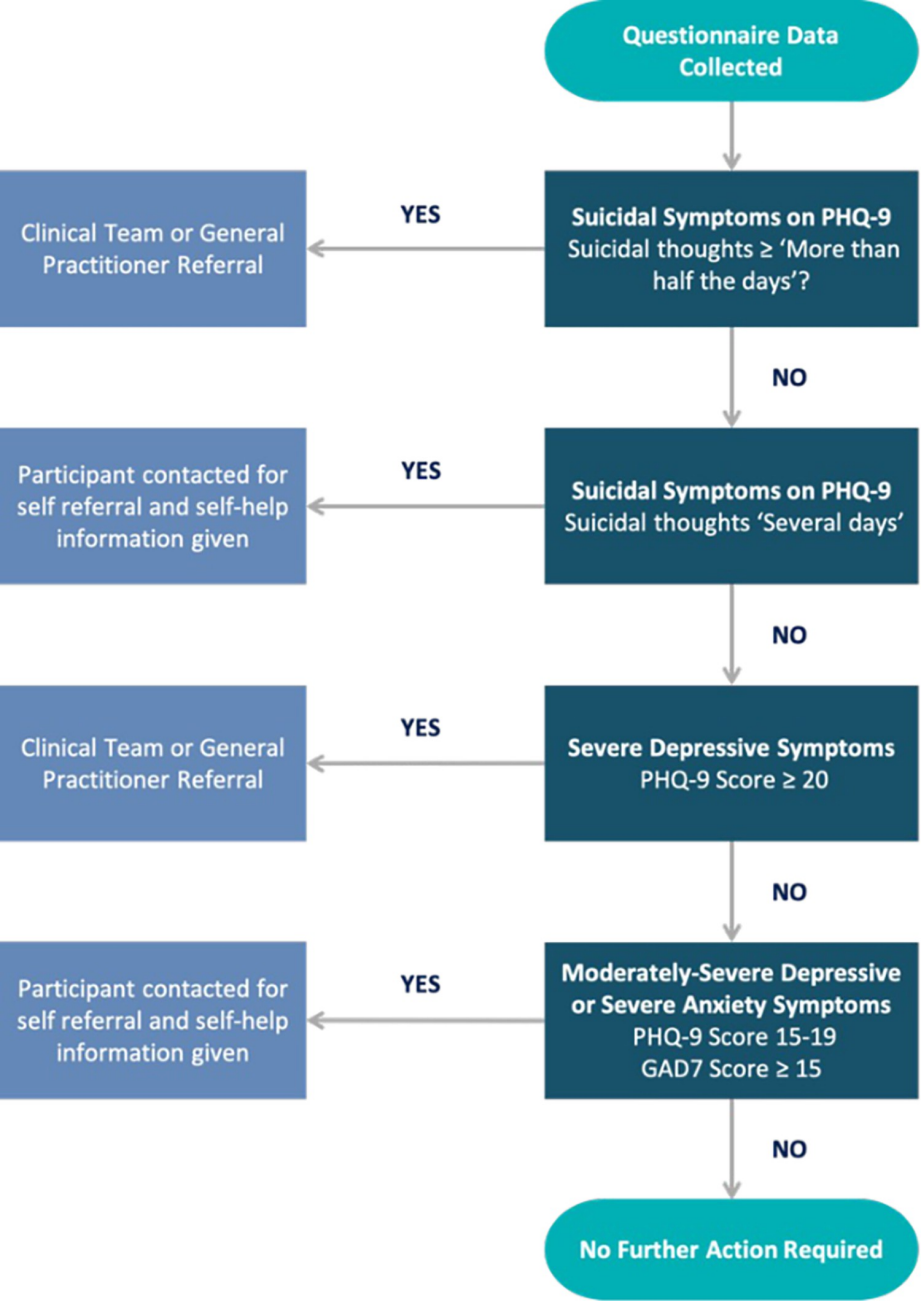
Decision tree for referral requirement post questionnaire completion.

While this observational study is low risk, questionnaires completed by participants cover sensitive topics around mental health, of which some cannot ethically be ignored if issues are identified. These include concerns surrounding severe depression, anxiety, and suicidal ideation. Whilst expected to be rare in the study population a protocol has been put into place to ensure issues of concern are appropriately reviewed by a relevant healthcare professional (Fig 3). Those with less severe symptoms will receive relevant information on where self-help can be gained and encouraged to self-refer to the general practitioner or local Improving Access to Psychological Therapies (IAPT) services. However, for more severe symptoms direct referral to the usual care team or the participants general practitioner is made. Participants are made aware of this requirement during the consenting process. Furthermore, within the study website links to well recognised information and help sources are provided, including official NHS websites, a link to the Samaritans website and a link to identify local IAPT services.

### Patient and public involvement (PPI)

Patient involvement was actively sought during the design of the study through its presentation at the South East London Consumer Research Panel for Cancer (SELCRP) at Guy’s Cancer centre, with a panel of 15 cancer patients. Feedback was sought on suitability and importance of research question, study design, consenting methods and patient burden of the study. Feedback received demonstrated the research question to be relevant and of high importance to the quality of life of cancer patients. The study burden was deemed small in view of minimal time requirement and ability to complete the questionnaires at home. Therefore, it was viewed patient involvement would overall benefit future cancer care. The telephone consenting method was an acceptable method for delivery of information, with virtual forms acceptable for those with access. The primary concern for study methodology, included accessibility and inclusivity of all patients for the remote study design. This was subsequently addressed by ensuring the availability of different methods of completion of the follow up questionnaires, either via postal or email links. Additionally, participants and the public will form part of the dissemination strategy. Participants will be informed of the study results on publication of results, with findings openly available on the study website and disseminated through social media.

## Discussion

MIND-P is a prospective cohort study that aims to primarily evaluate prognostic factors for mental wellbeing outcomes in newly diagnosed prostate cancer patients in the initial follow-up period. With the current paucity of data available for prognostic factors in these outcomes within a prostate cancer population, this study will mainly serve as an exploratory study to further identify potential patient, oncological and treatment factors of interest. These findings will be of importance by identifying factors that should subsequently be specifically evaluated in further prospective studies within different settings to identify their individual prognostic value [19]. Additionally, these findings will be of use when considering future prognostic model research within the field, helping to identify candidate predictor variables and limit the number of variables which are evaluated within an individual model [20]. Lastly, findings will be of use clinically. Not only may they highlight the prevalence of mental wellbeing issues in prostate cancer, but also could help identify individuals who may be at increased risk of developing issues, and thereby highlighting those who require additional monitoring or support.

Strengths of this study include its prospective longitudinal design with much of the existing literature evaluating these outcomes within cancer being largely cross-sectional. Additionally, unlike many studies, we are seeking to evaluate multiple relevant mental wellbeing outcomes, which were selected as important outcomes to prostate cancer patients through extensive background work. Thereby, this allows for a more holistic evaluation of mental wellbeing, rather than overly focused on pure mental health outcomes or specific individual constructs. However, as with any study potential limitations exist. Firstly, the observational methodology means that factors evaluated cannot be subsequently causally attributed to outcomes of interest. However, with the aim being to evaluate prognostic factors rather than causal evaluation this may not necessarily hinder study results. Additionally, whilst employing a multi-institutional design across multiple settings, findings for this study remain within the UK context and are limited to the recruited treatment groups with those undergoing chemotherapy or HIFU excluded. Lastly, the study is primarily powered for detecting differences between treatment cohorts meaning that findings for remaining patient and oncological factors should still be considered exploratory.

Dissemination of subsequent study results is aimed across several platforms. Firstly, presentation at local, national, and international meetings across urological, oncological, and psychological settings is planned. Additionally, final findings will be published within a peer-reviewed urology or oncology journal. Findings will also subsequently be distributed through social media of authors and the study website to ensure as widespread dissemination as possible. Lastly, findings will be directly distributed to participants as per the study PPI plan.

## Supporting information

S1 File Full participant information sheet (PIS) and consent form for study. (PDF)

S2 File Complete MIND-P questionnaire utilised for study data collection. (PDF)
