# Supplementary material for: Mental wellbeing and quality of life in prostate cancer (MIND-P): Protocol for a multi-institutional prospective cohort study
Source: PLoS One. 2023 Apr 24;18(4):e0284727. doi: 10.1371/journal.pone.0284727 (PMC10124830; doi:10.1371/journal.pone.0284727)
Supplement: S1 File — (PDF) [file pone.0284727.s001.pdf]

Participant information sheet

Mental wellbeIng aND quality of life in Prostate cancer (MIND-P)

PART 1

We'd like to invite you to take part in our research study.
Joining the study is entirely up to you, before you decide, we would like you to understand why the research is being done and what it would involve for you.
One of our team will go through this information sheet with you, to help you decide whether or not you would like to take part and answer any questions you may have. We estimate this should take about 20 minutes.
Please feel free to talk to others about the study if you wish.

The first part of the Participant Information Sheet tells you the purpose of the study and what will happen to you if you take part.

The second part will give you more detailed information about the conduct of the study. Please ask if anything is unclear.

What is the purpose of the study?

The purpose of this study is to explore the impact a prostate cancer diagnosis and its various treatments have on a patient's mental wellbeing and quality of life. The wellbeing and quality of life issues we are looking at include depressive and anxiety symptoms, body image issues, fear of recurrence, masculinity perception and functional issues such as bladder, bowel or sexual symptoms. More specifically, this study is aiming to look at how patients who undergo different management strategies for their cancer, experience these issues differently, and compare these groups directly. The four different treatment groups we are evaluating are:

- Active Surveillance
- Surgery (Radical Prostatectomy)
- Radiotherapy
- Hormonal Treatment

The reason for looking at this is because we want to better understand just how common these issues are in each treatment group, and which patients specifically are at greatest risk of having problems after a prostate cancer diagnosis. This would provide useful information for us on which patients may require further support during their cancer treatment.

This study is additionally being conducted as part of an educational project for a King's College London student in part fulfillment of the award of a PhD degree.

Why have I been invited?

You have been invited to take part in the study because you have been recently diagnosed with prostate cancer. Patients from eight hospitals will be invited to take part, including King's College Hospital (King's College Hospital NHS Foundation Trust), Princess Royal University Hospital

(King’s College Hospital NHS Foundation Trust), Guy’s Hospital (Guy's and St Thomas' NHS Foundation Trust), Medway Maritime Hospital (Medway NHS Foundation Trust), Charring Cross Hospital (Imperial College Healthcare NHS Trust), Queen Elizabeth Hospital (Lewisham and Greenwich NHS Trust), East Surrey Hospital (Surrey and Sussex Healthcare NHS Foundation Trust) and Bedford Hospital (Bedfordshire Hospitals NHS Foundation Trust). We expect approximately 300 patients will take part in this study.

Do I have to take part?

It is up to you to decide. We will describe the study and go through this information sheet, which we will then give to you. You will be able to keep this information sheet and think about taking part. You are free to discuss the information with anyone you wish including your family and friends. If you agree, we will then ask you to sign a consent form to show you have agreed to take part. You are free to withdraw at any time, without giving a reason. This would not affect the standard of care you receive.

What will happen to me if I take part?

If you agree to take part in the study, we will ask whether you prefer to have all paperwork and study follow ups done via email or post. A member of the research team will contact you using a telephone number given to us by your clinical team and go over the study in detail, allowing you to ask any questions. If you are still happy to take part, we will then ask you to sign either an electronic or a physical consent form which has been sent to you depending on your stated preference when you were recruited. A signed copy of either of these will then be provided to you after this using the same method used to complete the form (i.e posted or emailed to yourself).

This study is purely observational, where you will receive no intervention or treatment for your prostate cancer outside of what will be given to you by your clinical team. We will instead follow you up for a total of 12 months using a series of questionnaires sent to you. These will be sent to you electronically via email, or physically through the post depending on your preference. The questionnaires would then be sent to you at the beginning of the study and then a further four times at 3, 6, 9 and 12 months after your cancer diagnosis. The same questionnaire will be sent to you at each of these time frames. It is estimated that on each occasion the questionnaires will take approximately 30 minutes to complete. Therefore, it is estimated that a total of two and a half hours of your time will be required over the duration of the 12 months of this study. A copy of the questionnaire will be provided to you with this information leaflet as a supplement. You can use this for consideration before deciding whether you wish to take part in the study or not. The questionnaires will ask you about recent symptoms you have experienced including depressive and anxiety symptoms, feelings about your body image, fears of your disease coming back or getting worse, your perceptions around masculinity and physical symptoms you have experienced including bladder, bowel and sexual function. In addition to this at the beginning and at the end of the study, the research team will also gain some extra information specifically about your cancer and the treatments you have received from your clinical records.

There will be no need for any physical follow up at a hospital site for the purpose of this study. We will only follow you up using the methods above. All data collected from yourself during this study will use secure data collection methods, be stored safely and study staff will protect your personal information closely so no one will be able to connect your responses and any other information that identifies you. At the end of the 12 months your involvement in the study will finish with no requirement for further questionnaires and we will subsequently inform you of the results of the study.

What are the alternatives for treatment?
Taking part in this study will not alter the treatment or follow up you receive for your prostate cancer. All treatments you receive will still be managed by your clinical team as per their decisions.

What are the possible benefits of taking part?
As no treatment is being received during the study itself it is unlikely being involved will benefit your own cancer outcomes. We therefore cannot promise the study will help you personally. However, the information we get from this study may help improve the future treatment of people with prostate cancer by helping us to identify who is most likely to need help during their cancer experience.

What are the possible disadvantages and risks of taking part?
As we will not be giving you any additional treatment or intervention affecting your care in this study there are no side-effect related risks from participating in this study. However, a disadvantage from participating is the inconvenience and time associated with completing the necessary questionnaires which we expect will require around 30 minutes at each time point. We aim to minimise this by sending these to you (by whichever means you selected at the outset) to complete at a time convenient to you. Additionally, the questionnaires will ask sensitive questions surrounding your mental health and symptoms such as bowel, bladder and sexual function, which some find distressing to share. Throughout the study we will keep these results confidential, ensuring your data is also secure through our data storage policy.

Lastly, as we will be following you with mental health questionnaires which are not usually part of the routine follow up procedure, we may identify mental health problems which would not otherwise be discovered. Whilst having mild depressive or anxiety symptoms are common, more severe symptoms including thoughts of suicide may well be identified in a very small proportion of participants. We have made a protocol on what would happen if these were identified in any of our participants. If we were to see features of severe depression or frequent suicidal thoughts, we would discuss these with yourself, and importantly, with either your usual clinical team or your General Practitioner, so that help can be given to you if needed. For those where we identify less severe depressive or suicidal symptoms, or where severe anxiety was seen we will contact you with further information on ways to seek self-help or how to make a self-referral locally. We will additionally have further resources available within our study website and a phone contact if you are concerned and would like to discuss any issues further. Details of both of these are available at the end of this document.

Who is organising and funding this study?
The doctor in charge of this study is Mr. Kamran Ahmed, an honorary consultant urological surgeon at King's College Hospital and a Senior Clinical Lecturer at King's College London. The study is funded by King's College London through the King's Medical Research Trust. The sponsor of the study is King's College London, with King's College Hospital NHS Foundation Trust acting as a co-sponsor. Therefore, any reference to 'we' within this document refers to the study team and sponsors and not the local site. There are no payments made by us to your hospital/ doctor for including you in this study.

How have patients and the public been involved in this study?
In designing of this study, we have taken into account cancer patient opinions through discussion of the study at a South East London Consumer Research Panel for Cancer (SELCRP) meeting. The topic of research, questionnaires to be used, the frequency of sending questionnaires and the overall burden of the study were discussed with members of the panel. The feedback received has strengthened the view that this is an important topic during cancer care, has guided aspects of the design of the study and re-assured us that the burden of participation for those participating is seen as low overall in view of the potential benefits for future care.

Who has reviewed this study?
All research in the NHS is looked at by an independent group of people, called a Research Ethics Committee, to protect your interests. This study has been reviewed and given a favourable opinion by the London - Harrow Research Ethics Committee (REC Reference: 20/LO/1136). It has also been approved by the Health Research Authority and each local hospital will also give confirmation that the study can go ahead.

Expenses and Payments
As no costs are associated with participating in this study. There are no funds available for payments to those participating in this study. If you chose to participate through postal follow-up pre-paid envelopes will be provided for this purpose.

What happens when the research study stops?
At the end of the 12 months of follow up there will be no further need for you complete any further questionnaires. We will collect some final data from your clinical records at this point regarding your cancer and the treatment you received but you will not require to do anything further for this. We will subsequently inform you of the results of the study through the same method we have been following you up with (postal or email) and we will post the results of these on our website.

This completes Part 1 of the Information Sheet.

If the Information in Part 1 has interested you and you are considering participation, please continue to read the additional information in Part 2 before making any decision.

PART 2

What will happen if I don't want to carry on with the study?
You are free to withdraw from the study at any time; and if you would like to do so; please speak to any member of the research team. It is important to know that your decision to withdraw from the study will not affect any care you receive. If you withdraw your consent to participate, we will not send you any further questionnaires at future dates and we will not collect any further clinical data from your records.

If you withdraw your consent; De-identified information collected about you may be used if you are happy with this. You can however request for all information collected already during the study to be destroyed, provided this was still possible (i.e. provided your data has not been used for final analysis by this point)

Similarly, if you were to die during the 12 months you were being followed up for this study, we would contact your next of kin (as detailed in your medical records) and confirm that they are happy for us to use de-identified information collected about you up to that point. We would remove any information collected up to this point if your next of kin was not in agreement for this to be done.

What if there is a problem?
If you have a concern about any aspect of this study, you should ask to speak to a member of the research team who will do their best to answer your questions. The coordinating member of the research study (Mr. Oliver Brunckhorst) can be contacted through oliver.brunckhorst@kcl.ac.uk or through our dedicated study telephone on 07434672408 for the discussion of any issues. Alternatively, the responsible lead of the research project (Mr. Kamran Ahmed) can also contacted through kamran.ahmed@kcl.ac.uk. If you remain unhappy and wish to complain formally, you can do this through the NHS Complaints procedure by contacting your local Patient Advice Liaison Service (PALS) office. Details of your local office can be obtained by asking your study doctor, GP, telephoning your local hospital or looking on the NHS choices website. http://www.nhs.uk/pages/home.aspx

Every care will be taken in the course of this study. However in the unlikely event that you are injured by taking part, compensation may be available.

In the event that something does go wrong and you are harmed during the research and this is due to someone's negligence then you may have grounds for a legal action for compensation against King's College London, but you may have to pay your legal costs.

Regardless of this, if you wish to complain, or have any concerns about any aspect of the way you have been approached or treated by members of staff or about any side effects (adverse events) you may have experienced due to your participation in the study the normal National Health Service complaints mechanisms are available to you. Please ask a member of the research team if you would like more information on this.

Will my taking part be kept confidential?

Study staff will protect your personal information closely so no one will be able to connect your responses and any other information that identifies you individually. However, national laws may require us to show information to university or government officials (or sponsors), who are responsible for monitoring the safety of this study. Directly identifying information (e.g. names, addresses) will be safeguarded and maintained under controlled conditions. You will not be identified in any publication from this study.

The organisations listed above will keep information about you confidential and secure. Your name will not be used in any reports about the study and all data is stored in accordance with the principle of the Data Protection Act 2018 and General Data Protection Regulations (GDPR). The chief investigator (Mr Kamran Ahmed) will act as the data custodian for this study, ensuring access to the information is only to those directly involved in the research. King's College London will act as the data controllers of the study and is responsible for the security and validity of the information held under the Data Protection Act.

To ensure that the data we collect is secure during the study we will also:

- Store all data using secure password protected and encrypted devices which will be kept in safe locations at our King's College London research offices. These are located at Guy's Hospital and King's College Hospital.
- All identifiable data we have from you including your name, contact details and date of birth will be kept separately from questionnaire data we collect from you so that this information is not linked to the sensitive data we collect.
- All sensitive data we keep will be de-identified, where we replace any identifying information with unique identifying codes. Only the research team will have access to information that identifies you to carry out this research study. Your identifying information will not be shared with others outside this research study
- All data including questionnaire data or clinical data collected from you, which is collected using electronic survey software will use secure transfer and storage of data principles. No identifying features collected which are linked to the sensitive information you provide us.
- All postal data collected, which includes the questionnaire sent to you if this is your chosen method of follow up, will also not contain your identifiable information with only your study ID used to ensure we know who the survey results belong to.
- We will not send your data externally to be analysed and the data will not be stored or sent outside the European Economic Area (EEA)

All information gained from the questionnaires will remain confidential and only used for subsequent analysis of data by members of the research team. However, as previously mentioned if during the follow up we identify any severe mental health concerns about you where we are severely concerned about your safety (e.g. severe depression or frequent suicidal thoughts), we would subsequently contact your usual hospital team or your GP to discuss these concerns with them. We would discuss this with you prior and this would only be done so that any concerns could then be addressed by a suitable healthcare professional.

How will we use information about you?

If you consent to take part, we will need to use information from you for conducting the research project. This will include your contact information, data we collect from your clinical records about yourself, your cancer and the treatment you have received, and the information you provide from the questionnaires we send you. This data will only be used to contact you during the study and for the analysis of the data to meet our research aims by members of the research team. As mentioned before de-identified data have to be shared to regulatory bodies to ensure the research is being done properly, however your personal information will be safeguarded.

Once the study has completed some of the data is required to be kept for 20 years as per King's College London policy. We will keep identifying information only for a period of 1 year after the completion of the study, purely for the purpose of informing you of the results of the study. After this we will destroy this information. After this all data we keep will not contain any personal information that could identify you. This de-identified data will be kept for a minimum of 20 years after completion of the study. The de-identified data will subsequently be made publicly available through King's College London data sharing website for the duration of the time it is kept. The purpose of this is purely to ensure that research is open to peer scrutiny, to optimise the use of good quality research data and to support policy and other decision-making. We do not plan to re-analyse this data during this period or apply for further ethics to re-use the data in any way. In addition, we will write any subsequent reports or publications arising from this study in a way that no-one can work out that you took part in the study.

What are your choices about how your information is used?

You can stop being part of the study at any time, without giving a reason, but we will keep de-identified information about you already collected to that point unless you request these to be withdrawn. Because we need to manage your records in specific ways for the research to be reliable, you can request to see the data we have about you (at your request), however, you won't be able change the data we hold.

Where can you find out more about how your information is used?

If you consent to take part, we will use your data to deliver this project as described in the Patient Information Sheet. If you would like to find out more please read the supplementary leaflet provided, entitled 'How we use your data'. Alternatively, you can find out more by asking one of the research team directly or by using the contact details at the end of this information sheet. Additionally, the Data Protection Officer for the Sponsor of the research (King's College London) is Mr Albert Chan who can be contacted through info-compliance@kcl.ac.uk should you have any concerns about how your data is handled.

Involvement of the General Practitioner/Family Doctor (GP)

If you agree to participate in this study, we will send your GP a letter to inform them of your participation in this study and what this involves. We will seek your permission for this to be done. Additionally, as previously mentioned, if during the conductance of the study any urgent mental health concerns arise, we would liaise with your GP or usual team to discuss these.

What will happen to the results of the research study?

We are hoping to publish the results of the study in peer reviewed journals and present these results at national and international conferences. We will also be publishing the results of the study in our website and on social media once these are published to ensure they are spread as widely as possible. The study will also form part of a research student PhD thesis as part of their degree. We will also be sharing the results of the study with the participants so that you are aware what the findings of the research are. As previously mentioned, there will be no identifiable information in any of the reports or publications we write from this study.

Thank you

Thank you for considering taking part and taking the time to read this information sheet.
If you decide to take part in the study, we will give you a copy of the information sheet and a signed consent form to keep.

Further information and contact details

If you would like further information regarding the study, please do not hesitate to ask one of the members of the research team directly or through our study phone. They will be happy to answer any queries or direct you to the right individuals. Additionally, we have further information within our study website and where to seek help if you require this:

Chief Investigator: Mr Kamran Ahmed, Tel: +44(0)20 7188 5906, Email: Kamran.ahmed@kcl.ac.uk
PhD Fellow/study coordinator: Mr Oliver Brunckhorst, Email: oliver.brunckhorst@kcl.ac.uk
MIND-P Research Team Contact Telephone Number: 07434672408 (Available Mon-Fri, 9:00-17:00)
MIND-P Study Website: www.kclmind-u.org.uk/the-mind-p-study/

[redacted]

This document explains how health researchers use information from patients. If you are asked to take part in research, you can ask what will happen in the study.

What is patient data?

When you go to your GP or hospital, the doctors and others looking after you will record information about your health. This will include your health problems, and the tests and treatment you have had. They might want to know about family history, if you smoke or what work you do. All this information that is recorded about you is called patient data or patient information.

When information about your health care joins together with information that can show who you are (like your name or NHS number) it is called identifiable patient information. It's important to all of us that this identifiable patient information is kept confidential to the patient and the people who need to know relevant bits of that information to look after the patient. There are special rules to keep confidential patient information safe and secure.

What sort of patient data does health and care research use?

There are lots of different types of health and care research.

If you take part in a clinical trial, researchers will be testing a medicine or other treatment. Or you may take part in a research study where you have some health tests or answer some questions. When you have agreed to take part in the study, the research team may look at your medical history and ask you questions to see if you are suitable for the study. During the study you may have blood tests or other health checks, and you may complete questionnaires. The research team will record this data in special forms and combine it with the information from everyone else in the study. This recorded information is research data.

In other types of research, you won’t need to do anything different, but the research team will be looking at some of your health records. This sort of research may use some data from your GP, hospital or central NHS records. Some research will combine these records with information from other places, like schools or social care. The information that the researcher collects from the health records is research data.

Why does health and care research use information from patients?

In clinical trials, the researchers are collecting data that will tell them whether one treatment is better or worse than other. The information they collect will show how safe a treatment is, or whether it is making a difference to your health. Different people can respond differently to a treatment. By collecting information from lots of people, researchers can use statistics to work out what effect a treatment is having.

Other types of research will collect data from lots of health records to look for patterns. It might be looking to see if any problems happen more in patients taking a medicine. Or to see if people who have screening tests are more likely to stay healthier.

Some research will use blood tests or samples along with information about the patient’s health. Researchers may be looking at changes in cells or chemicals due to a disease.

All research should only use the patient data that it really needs to do the research. You can ask what parts of your health records will be looked at.

How does research use patient data?

If you take part in some types of research, like clinical trials, some of the research team will need to know your name and contact details so they can contact you about your research appointments, or to send you questionnaires. Researchers must always make sure that as few people as possible can see this sort of information that can show who you are.

In lots of research, most of the research team will not need to know your name. In these cases, someone will remove your name from the research data and replace it with a code number. This is called coded data, or the technical term is

pseudonymised data. For example, your blood test might be labelled with your code number instead of your name. It can be matched up with the rest of the data relating to you by the code number.

In other research, only the doctor copying the data from your health records will know your name. They will replace your name with a code number. They will also make sure that any other information that could show who you are is removed. For example, instead of using your date of birth they will give the research team your age. When there is no information that could show who you are, this is called anonymous data.

Where will my data go?

Sometimes your own doctor or care team will be involved in doing a research study. Often, they will be part of a bigger research team. This may involve other hospitals, or universities or companies developing new treatments. Sometimes parts of the research team will be in other countries. You can ask about where your data will go. You can also check whether the data they get will include information that could show who you are. Research teams in other countries must stick to the rules that the UK uses.

All the computers storing patient data must meet special security arrangements.

If you want to find out more about how companies develop and sell new medicines, the Association of the British Pharmaceutical Industry has information on its website.

What are my choices about my patient data?

- You can stop being part of a research study at any time, without giving a reason, but the research team will keep the research data about you that they already have. You can find out what would happen with your data before you agree to take part in a study.
- In some studies, once you have finished treatment the research team will continue to collect some information from your doctor or from central NHS records over a few months or years so the research team can track your health. If you do not want this to happen, you can say you want to stop any more information being collected.

- Researchers need to manage your records in specific ways for the research to be reliable. This means that they won’t be able to let you see or change the data they hold about you. Research could go wrong if data is removed or changed.

What happens to my research data after the study?

Researchers must make sure they write the reports about the study in a way that no-one can work out that you took part in the study.

Once they have finished the study, the research team will keep the research data for several years, in case they need to check it. You can ask about who will keep it, whether it includes your name, and how long they will keep it.

Usually your hospital or GP where you are taking part in the study will keep a copy of the research data along with your name. The organisation running the research will usually only keep a coded copy of your research data, without your name included. This is kept so the results can be checked.

If you agree to take part in a research study, you may get the choice to give your research data from this study for future research. Sometimes this future research may use research data that has had your name and NHS number removed. Or it may use research data that could show who you are. You will be told what options there are. You will get details if your research data will be joined up with other information about you or your health, such as from your GP or social services.

Once your details like your name or NHS number have been removed, other researchers won’t be able to contact you to ask you about future research. Any information that could show who you are will be held safely with strict limits on who can access it.

You may also have the choice for the hospital or researchers to keep your contact details and some of your health information, so they can invite you to take part in future clinical trials or other studies. Your data will not be used to sell you anything. It will not be given to other organisations or companies except for research.

Will the use of my data meet GDPR rules?

GDPR stands for the General Data Protection Regulation. In the UK we follow the GDPR rules and have a law called the Data Protection Act. All research using patient data must follow UK laws and rules.

Universities, NHS organisations and companies may use patient data to do research to make health and care better. When companies do research to develop new treatments, they need to be able to prove that they need to use patient data for the research, and that they need to do the research to develop new treatments. In legal terms this means that they have a 'legitimate interest' in using patient data.

Universities and the NHS are funded from taxes and they are expected to do research as part of their job. They still need to be able to prove that they need to use patient data for the research. In legal terms this means that they use patient data as part of 'a task in the public interest'. If they could do the research without using patient data they would not be allowed to get your data.

Researchers must show that their research takes account of the views of patients and ordinary members of the public. They must also show how they protect the privacy of the people who take part. An NHS research ethics committee checks this before the research starts.

What if I don't want my patient data used for research?

You will have a choice about taking part in a clinical trial testing a treatment. If you choose not to take part, that is fine.

In most cases you will also have a choice about your patient data being used for other types of research. There are two cases where this might not happen:

1. When the research is using anonymous information. Because it's anonymous, the research team don't know whose data it is and can't ask you.
2. When it would not be possible for the research team to ask everyone. This would usually be because of the number of people who would have to be contacted. Sometimes it will be because the research could be biased if some people chose not to agree. In this case a special NHS group will check that the reasons are valid. You can opt-out of your data being used for this sort of research. You can ask your GP about opting-out, or you can find out more.

Who can I contact if I have a complaint?

If you want to complain about how researchers have handled your information, you should contact the research team. If you are not happy after that, you can contact the Data Protection Officer. The research team can give you details of the right Data Protection Officer.

If you are not happy with their response or believe they are processing your data in a way that is not right or lawful, you can complain to the Information Commissioner's Office (ICO) (www.ico.org.uk or 0303 123 1113).

Participant Consent Form

Mental wellbeIng aND quality of life in Prostate cancer (MIND-P)

A prospective and longitudinal cohort study assessing Mental wellbeIng aND quality of life in Prostate cancer – the MIND-P Study

IRAS Number: 284473
Chief Investigator: Mr Kamran Ahmed
Participant Identification Number for this study:
Participant Date of Birth:

		Please initial box
**1**	I confirm that I have read the information sheet dated 09.02.2022 (Version 3.0) for the above study. I have had the opportunity to consider the information, ask questions and have had these answered satisfactorily.	☐
**2**	I understand that my participation is voluntary and that I am free to withdraw at any time without giving any reason, without my medical care or legal rights being affected.	☐
**3**	I understand that taking part in this study involves completing questionnaires about mental wellbeing and symptoms experienced on five separate occasions	☐
**4**	I agree to give members of the research team access to relevant parts of my hospital medical records to extract relevant data about myself, my condition and treatments I have received.	☐
**5**	I understand that relevant sections of my medical notes and data collected during the study, may be looked at by individuals from the sponsor of the study (King's College London) and responsible persons authorised by the sponsor, from regulatory authorities or from the NHS Trust, where it is relevant to my taking part in this research. I give permission for these individuals to have access to my records.	☐
**6**	I understand that personal information will be collected about me that can identify me, such as my name and contact details, so that the research team can follow me up during the study. I understand these will not be shared beyond the study team.	☐
**7**	I understand that information I provide and is collected will be analysed and used for future research publications by the research team.	☐
**8**	I consent for my GP to be informed of my participation in this study	☐
**9**	I understand that if during the study follow up any severe mental health concerns were to arise; the research team would subsequently discuss these with your GP or usual hospital team.	☐

10 I give permission for the de-identified (anonymised) collected data from this study to be deposited in a publicly data repository, so it can be used for transparency, future research, and learning. ☐

11 I voluntarily consent to be a participant in the above study. ☐

____________________	____________________	____________________
Name of Participant	Date	Signature

____________________	____________________	____________________
Name of Person taking consent	Date	Signature

When completed: 1 for participant and 1 for researcher site file
