# Supplementary material for: Mental wellbeing and quality of life in prostate cancer (MIND-P): Protocol for a multi-institutional prospective cohort study
Source: PLoS One. 2023 Apr 24;18(4):e0284727. doi: 10.1371/journal.pone.0284727 (PMC10124830; doi:10.1371/journal.pone.0284727)
Supplement: S2 File — (PDF) [file pone.0284727.s002.pdf]

Mental wellbeIng aND quality of life in Prostate cancer (MIND-P)

Study Questionnaire

IRAS Number: 284473

Chief Investigator: Mr Kamran Ahmed

Participant Identification Number:

Questionnaire Number:

Treatment Information

Please circle your response to the following questions about any recent treatment received for your prostate cancer or mental health

1. Since your last questionnaire have you undergone any further treatment for your prostate cancer? Y / N
   If Yes, please provide further detail of what treatment was received below:

2. Have you started any new treatment for depression or anxiety since your last questionnaire? Y / N
   If Yes, what treatment e.g. Anti-depressant medication or cognitive behavioural therapy:

3. Have you required any admissions into hospital or another institution directly because of depression, anxiety or other mental health concerns since your last questionnaire? Y / N
   If Yes, please provide further information:

Patient Health Questionnaire-9 Scale (PHQ9)

Over the **last two weeks** how often have you been bothered by the following problems?		0<br>Not at all	1<br>Several days	2<br>More than half the days	3<br>Nearly every day
1	Little interest or pleasure in doing things	☐	☐	☐	☐
2	Feeling down, depressed, or hopeless	☐	☐	☐	☐
3	Trouble falling or staying asleep, sleeping too much	☐	☐	☐	☐
4	Feeling tired or having little energy	☐	☐	☐	☐
5	Poor appetite or overeating	☐	☐	☐	☐
6	Feeling bad about yourself – or that you are a failure or have let yourself or your family down	☐	☐	☐	☐
7	Trouble concentrating on things, such as reading the newspaper or watching television	☐	☐	☐	☐
8	Moving or speaking so slowly that other people could have noticed. Or the opposite – being so fidgety or restless that you have been moving around a lot more than usual	☐	☐	☐	☐
9	Thoughts that you would be better off dead or of hurting yourself in some way	☐	☐	☐	☐

Generalised Anxiety Disorder – 7 Scale (GAD7)

Over the **last two weeks** how often have you been bothered by the following problems?		0<br>Not at all	1<br>Several days	2<br>More than half the days	3<br>Nearly every day
1	Feeling nervous, anxious, or on edge	☐	☐	☐	☐
2	Not being able to stop or control worrying	☐	☐	☐	☐
3	Worrying too much about different things	☐	☐	☐	☐
4	Trouble relaxing	☐	☐	☐	☐
5	Being so restless that it's hard to sit still	☐	☐	☐	☐
6	Becoming easily annoyed or irritable	☐	☐	☐	☐
7	Feeling afraid as if something awful might happen	☐	☐	☐	☐

Body Image Scale

In this questionnaire you will be asked how you feel about your appearance, and about any changes than may have resulted from your disease or treatment.

Please read each item carefully, and place a firm tick on the box alongside the reply which comes closest to the way you have been feeling about yourself, **during the past week**		Not at all	A little	Quite a bit	Very much
1	Have you been feeling self-conscious about your appearance	☐	☐	☐	☐
2	Have you felt less physically attractive as a result of your disease or treatment?	☐	☐	☐	☐
3	Have you been dissatisfied with your appearance when dressed?	☐	☐	☐	☐
4	Have you been feeling less masculine as a result of your diseases or treatment?	☐	☐	☐	☐
5	Did you find it difficult to look at yourself naked?	☐	☐	☐	☐
6	Have you been feeling less sexually attractive as a result of your disease or treatment?	☐	☐	☐	☐
7	Did you avoid people because of the way you felt about your appearance	☐	☐	☐	☐
8	Have you been feeling the treatment has left your body less whole?	☐	☐	☐	☐
9	Have you felt dissatisfied with your body?	☐	☐	☐	☐

Fear of Cancer Recurrence Scale-7 (FCR7)

Please answer the following questions by placing a tick in any of the boxes for each of the question.		1	2	3	4	5
		Not at all	A little	Sometimes	A lot	All the time
1	I am afraid that my cancer may recur or progress	☐	☐	☐	☐	☐
2	I am worried or anxious about the possibility of cancer recurrence or progression	☐	☐	☐	☐	☐
3	How often have you worried about the possibility of getting cancer again or your cancer getting worse	☐	☐	☐	☐	☐
4	I get waves of strong feelings about the cancer coming back or getting worse	☐	☐	☐	☐	☐
5	I think about the cancer returning or getting worse when I didn't mean to	☐	☐	☐	☐	☐
6	I examine myself to see if I have physical signs of cancer	☐	☐	☐	☐	☐

		Not al all										A great deal
7	To what extent does worry about getting cancer again or it getting worse spill over or intrude on your thoughts and activities	0 ☐	1 ☐	2 ☐	3 ☐	4 ☐	5 ☐	6 ☐	7 ☐	8 ☐	9 ☐	10 ☐

Prostate Cancer-Related Quality of Life (PC-QOL)– Masculine Self Esteem Subset

How true has each of the following statements been for you during the **past 4 weeks?**		5<br>Not at all	4<br>A little bit	3<br>Somewhat	2<br>Quite a lot	1<br>Very much
1	I feel as if I am no longer a whole man	☐	☐	☐	☐	☐
2	I feel like I've lost part of my manhood	☐	☐	☐	☐	☐
3	I'm not the man I used to be	☐	☐	☐	☐	☐
4	I feel that others think that I'm not the man I used to be	☐	☐	☐	☐	☐
5	I feel weak and small	☐	☐	☐	☐	☐
6	I worry about being compared unfavourably to other men	☐	☐	☐	☐	☐
7	I feel I have been too emotional	☐	☐	☐	☐	☐
8	It's hard to think things through coolly and logically	☐	☐	☐	☐	☐

Functional Assessment of Cancer Therapy – General (FACT-G) Social/family well-being subscale

Please tick one number per line to indicate your response as it applies to the **past 7 days.**		0<br>Not at all	1<br>A little bit	2<br>Somewhat	3<br>Quite a bit	4<br>Very much
1	I feel close to my friends	☐	☐	☐	☐	☐
2	I get emotional support from my family	☐	☐	☐	☐	☐
3	I get support from my friends	☐	☐	☐	☐	☐
4	My family has accepted my illness	☐	☐	☐	☐	☐
5	I am satisfied with family communication about my illness	☐	☐	☐	☐	☐
6	I feel close to my partner (or the person who is my main support)	☐	☐	☐	☐	☐
7	I am satisfied with my sex life	☐	☐	☐	☐	☐

The Expanded Prostate Cancer Index Composite-26 (EPIC-26)

1. Over the **past 4 weeks**, how often have you leaked urine?

   More than once a day..................... 1
   About once a day............................ 2
   More than once a week.................... 3 (Circle one number)
   About once a week...........................4
   Rarely or never............................... 5

2. Which of the following best describes your urinary control **during the last 4 weeks**?

   No urinary control whatsoever........... 1
   Frequent dribbling.. 2 (Circle one number)
   Occasional dribbling... 3
   Total control.. 4

3. How many pads or adult diapers per day did you usually use to control leakage **during the last 4 weeks**?

   None 0
   1 pad per day.. 1
   2 pads per day.. 2 (Circle one number)
   3 or more pads per day... 3

4. How big a problem, if any, has each of the following been for you **during the last 4 weeks**?

   (Circle one number on each line)

	No Problem	Very Small Problem	Small Problem	Moderate Problem	Big Problem
a. Dripping or leaking urine	0	1	2	3	4
b. Pain or burning on urination.....	0	1	2	3	4
c. Bleeding with urination............	0	1	2	3	4
d. Weak urine stream or incomplete emptying	0	1	2	3	4
e. Need to urinate frequently during the day.....................................	0	1	2	3	4

5. Overall, how big a problem has your urinary function been for you **during the last 4 weeks**?

   No problem..................................... 1
   Very small problem.......................... 2
   Small problem.................................3 (Circle one number)
   Moderate problem........................... 4
   Big problem..................................... 5

6. How big a problem, if any, has each of the following been for you? (Circle one number on each line)

	No Problem	Very Small Problem	Small Problem	Moderate Problem	Big Problem
a. Urgency to have a bowel movement	0	1	2	3	4
b. Increased frequency of bowel movements.....................	0	1	2	3	4
c. Losing control of your stools......	0	1	2	3	4
d. Bloody stools	0	1	2	3	4
e. Abdominal/ Pelvic/Rectal pain...	0	1	2	3	4

7. Overall, how big a problem have your bowel habits been for you **during the last 4 weeks**?

No problem...................................... 1
Very small problem.......................... 2
Small problem.................................. 3 (Circle one number)
Moderate problem........................... 4
Big problem..................................... 5

8. How would you rate each of the following **during the last 4 weeks**? (Circle one number on each line)

	Very Poor to None	Poor	Fair	Good	Very Good
a. Your ability to have an erection?................................	1	2	3	4	5
b. Your ability to reach orgasm (climax)?......................	1	2	3	4	5

9. How would you describe the usual QUALITY of your erections **during the last 4 weeks**?

None at all.. 1
Not firm enough for any sexual activity.. 2
Firm enough for masturbation and foreplay only............................... 3 (Circle one number)
Firm enough for intercourse.. 4

10. How would you describe the FREQUENCY of your erections **during the last 4 weeks**?

I NEVER had an erection when I wanted one.................................. 1
I had an erection LESS THAN HALF the time I wanted one............. 2
I had an erection ABOUT HALF the time I wanted one .... 3 (Circle one number)
I had an erection MORE THAN HALF the time I wanted one............ 4
I had an erection WHENEVER I wanted one..................................... 5

11. Overall, how would you rate your ability to function sexually **during the last 4 weeks**?

Very poor..	1	
Poor..	2	
Fair...	3	(Circle one number)
Good...	4	
Very good..	5	

12. Overall, how big a problem has your sexual function or lack of sexual function been for you **during the last 4 weeks**?

No problem..	1	
Very small problem..	2	
Small problem..	3	(Circle one number)
Moderate problem..	4	
Big problem...	5	

13. How big a problem **during the last 4 weeks**, if any, has each of the following been for you? (Circle one number on each line)

	No Problem	Very Small Problem	Small Problem	Moderate Problem	Big Problem
a. Hot flashes…………............................	0	1	2	3	4
b. Breast tenderness/enlargement..	0	1	2	3	4
c. Feeling depressed…...................	0	1	2	3	4
d. Lack of energy..........…................	0	1	2	3	4
e. Change in body weight…………..	0	1	2	3	4

Short Form-12 (SF12)

1. In general, would you say your health is:

Excellent................................ 1
Very good............................. 2
Good.................................... 3
Fair....................................... 4
Poor...................................... 5

(Circle one number)

2. The following questions are about activities you might do during a typical day. Does your health now limit you in these activities? If so, how much?

(Circle 1, 2, or 3 on each line)

	Yes Limited A Lot	Yes, Limited A Little	No, Not Limited At All
a. **Moderate activities**, such as moving a table, pushing a vacuum cleaner, bowling, or playing golf……………	1	2	3
b. Climbing **several** flights of stairs………………………	1	2	3

3. During the PAST 4 WEEKS, have you had any of the following problems with your work or other regular daily activities as a result of your PHYSICAL HEALTH?

(Please answer YES or NO for each question by circling 1 or 2 on each line.)

	Yes	No
a. **Accomplished less** than you would like	1	2
b. Were limited in the **kind** of work or other activities	1	2

4. During the PAST 4 WEEKS, have you had any of the following problems with your work or other regular daily activities as a result of any EMOTIONAL PROBLEMS, such as feeling depressed or anxious?

(Please answer YES or NO for each question by circling 1 or 2 on each line)

	Yes	No
a. **Accomplished less** than you would like	1	2
b. Didn't do work or other activities as **carefully** as usual	1	2

5. During the past 4 weeks, how much did pain interfere with your normal work (including both work outside the home and housework)?

Not at all 1
Slightly 2
Moderately 3
Quite a bit 4
Extremely 5

(Circle one number)

6. These questions are about how you feel and how things have been with you during the PAST 4 WEEKS. For each question, please give the one answer that comes closest to the way you have been feeling. How much of the time during the past 4 weeks...

(Circle one number on each line)

	All of the Time	Most of the Time	A Good Bit of the Time	Some of the Time	A Little of the Time	None of the Time
a. Have you felt calm and peaceful?.....................	1	2	3	4	5	6
b. Did you have a lot of energy?	1	2	3	4	5	6
c Have you felt downhearted and blue?	1	2	3	4	5	6

7. During the past 4 weeks, how much of the time has your physical health or emotional problems interfered with your social activities (like visiting with friends, relatives, etc.)?

All of the time 1
Most of the time.........2
Some of the time.......3
A little of the time...... 4
None of the time........5

(Circle one number)

END OF QUESTIONNAIRES

THANK YOU VERY MUCH FOR YOUR TIME

PLEASE POST ALL COMPLETED PAGES BACK TO US USING THE PRE-PAID ENVELOPE PROVIDED

IF THERE ARE ANY PROBLEMS PLEASE DO NOT HESITATE TO CONTACT US
